# A Graphic Method for Identification of Novel Glioma Related Genes

**DOI:** 10.1155/2014/891945

**Published:** 2014-06-23

**Authors:** Yu-Fei Gao, Yang Shu, Lei Yang, Yi-Chun He, Li-Peng Li, GuaHua Huang, Hai-Peng Li, Yang Jiang

**Affiliations:** ^1^Department of Surgery, China-Japan Union Hospital of Jilin University, Changchun 130033, China; ^2^State Key Laboratory of Medical Genomics, Institute of Health Sciences, Shanghai Jiaotong University School of Medicine and Shanghai Institutes for Biological Sciences, Chinese Academy of Sciences, Shanghai 200025, China; ^3^Endoscopy Center, China-Japan Union Hospital of Jilin University, Changchun 130033, China; ^4^Institute of Systems Biology, Shanghai University, Shanghai 200444, China; ^5^CAS-MPG Partner Institute for Computational Biology, Shanghai Institutes for Biological Sciences, Chinese Academy of Sciences, Shanghai 200031, China

## Abstract

Glioma, as the most common and lethal intracranial tumor, is a serious disease that causes many deaths every year. Good comprehension of the mechanism underlying this disease is very helpful to design effective treatments. However, up to now, the knowledge of this disease is still limited. It is an important step to understand the mechanism underlying this disease by uncovering its related genes. In this study, a graphic method was proposed to identify novel glioma related genes based on known glioma related genes. A weighted graph was constructed according to the protein-protein interaction information retrieved from STRING and the well-known shortest path algorithm was employed to discover novel genes. The following analysis suggests that some of them are related to the biological process of glioma, proving that our method was effective in identifying novel glioma related genes. We hope that the proposed method would be applied to study other diseases and provide useful information to medical workers, thereby designing effective treatments of different diseases.

## 1. Introduction

Glioma is the most common and lethal intracranial tumor. It always revealed itself as malignant glioma which is usually divided into astrocytoma, oligodendroglioma, and oligoastrocytoma. Besides the classification based on histopathological features, glioma could also be graded on a WHO consensus-derived scale of I to IV by means of the degree of malignancy [1]. Clinically, most of the gliomas are high-grade gliomas (HGG). Glioblastoma (GBM), one of the HGG, accounts for more than half of gliomas [1]. Although the knowledge of glioma, especially HGG, has increased dramatically in recent years, many questions are still waiting for further elucidation. On the other hand, the overall 5-year survival rate of GBM remains less than 5% despite the advances in surgery, radiation, and chemotherapy [2].

In the previous reports, glioma always manifested itself with disordered pathways which regulated proliferation, survival, invasion, and angiogenesis. Among these biological processes, RB and p53 pathways are more inclined to be dysfunctional in GBM, and the disrepair could lead to the destruction of cell cycle by regulating the G1-to-S-phase transition [3, 4]. Furthermore, other pathways such as MAPK, PI3K/PTEN/AKT, and NF-*κ*B pathway are also overactivated in glioma and contribute to the uncontrollable cellular proliferation [5–7]. As we know, the tumorigenesis of glioma is a complicated process which involved intricate pathways beyond the above ones. To widely understand the mechanism underlying this disease, identification of its related genes and uncovering the relationship of them and the biological process of glioma are very important. However, it is time-consuming and expensive to identify novel glioma related genes by conventional experiments. On the other hand, encouraged by the successful application of computational methods to deal with various biological problems such as drug design [8–13] and analysis of complicated biological pathway [14–17], computational methods may address this problem and provide some useful information for investigators.

In this study, we proposed a graphic method and attempted to apply this method to discover novel glioma related genes. The current known glioma related genes collected from various sources were the firsthand information. Based on these genes, some new discovered genes were obtained by the well-known shortest path algorithm. Furthermore, a permutation test was conducted to exclude false positives among them. The analysis of the final remaining genes suggests that some of them had direct or indirect relationship to the biological process of glioma, indicating that this method was effective and may give new insight to study other diseases.

## 2. Materials and Methods

### 2.1. Materials

The current known glioma related genes were retrieved from the following sources. (1) All the 11 data sheets listed on the web page of COSMIC (http://cancer.sanger.ac.uk/cancergenome/projects/census/) were downloaded, from which we obtained 18 glioma related genes; (2) search for human diseases in UniProt (http://www.uniprot.org/) with keywords “human glioma oncogene” and “human glioma suppressor gene,” thereby obtaining 49 and 32 genes (only reviewed genes were selected), respectively; (3) select “Literature Search” in TSGene (http://bioinfo.mc.vanderbilt.edu/TSGene/search.cgi) and input “glioma” as keyword, obtaining 7 genes. After collecting all of the genes mentioned above, we finally obtained 77 glioma related genes, which were available in Supplementary Material I available online at http://dx.doi.org/10.1155/2014/891945.

### 2.2. Construction of a Weighted Graph from Protein-Protein Interactions

Protein-protein interaction (PPI) is useful information for investigating various biological problems [18–22]. Many computational methods were proposed based on the fact that proteins that can interact with each other always share similar functions. Since the known glioma related genes must have some common features related to glioma, it is reasonable to discover novel glioma related genes based on protein-protein interaction and known glioma related genes. The data concerning protein-protein interactions was downloaded from STRING (Search Tool for the Retrieval of Interacting Genes/Proteins, http://string.embl.de/) [23], a large database containing known and predicted protein interactions which are derived from genomic context, high-throughput experiments, (Conserved) Coexpression and Previous knowledge. In the obtained file, we extracted all protein-protein interactions of human. Each obtained interaction consists of two proteins and a score with range between 150 and 999, which can quantify the likelihood that an interaction may occur. For later formulation, let *Q*(*p*_1_, *p*_2_) denote the score of the interaction between two proteins *p*_1_ and *p*_2_. The constructed graph took proteins occurring at least one protein-protein interaction of human as nodes, while two nodes were adjacent if and only if the score of the interaction between the corresponding proteins was greater than zero. The obtained graph consisted of 18,600 nodes and 1,640,707 edges. Furthermore, to correctly reflect the strength of the interaction, each edge with nodes *v*_1_ and *v*_2_ was labeled by a weight, which can be computed by

(1) $$\begin{array}{c} W\left(v_{1},v_{2}\right)=1000-Q\left(p_{1},p_{2}\right), \end{array}$$

where *p*_1_ and *p*_2_ were two corresponding proteins of nodes *v*_1_ and *v*_2_, respectively.

### 2.3. Selection of Candidate Genes

**Figure 1:**
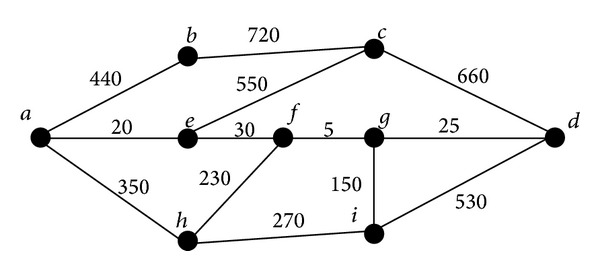
A simple example of the weighted graph.

It is obvious that the glioma related genes must have some common features which are related to glioma. On the other hand, as mentioned in Section 2.2, two proteins that can interact with each other, that is, they are adjacent in the constructed weighted graph, always share common features. The idea of our method was based on these facts. To clearly elaborate the idea of the method, we constructed a simple weighted graph which is shown in Figure 1, because the original graph was too large to exhibit in the paper. It is easy to observe from Figure 1 that the shortest path connecting *a* and *d* contains *e*, *f* and *g* as the inner nodes. Based on the weights of edges on this path, we can obtain that genes *a* and *e* can share common functions with high probability, because the confidence score of the interaction between *a* and *e* is very high, which is 1000 − 20 = 980. The similar results also hold for *e* and *f*, *f* and *g*, *g* and *d*. If genes *a* and *d* are two known glioma related genes, genes *e*, *f*, and *g* are actual glioma related genes with high likelihood. In view of this, shortest paths between any pair of known glioma related genes obtained by Dijkstra's algorithm, the most famous shortest path algorithm proposed by Dijkstra in 1956 [24], are useful information for further investigation.

After obtaining the shortest paths connecting any pair of known glioma related genes, it can be seen that some nodes/genes occurred in many paths, while most of nodes/genes in the graph were not contained in any path. Thus, for each node/gene in the graph, we counted the number of paths containing the node/gene, termed as betweenness which is defined as the number of shortest paths containing the node/gene as an inner node. The concept of betweenness has been employed in some studies of natural and man-made networks [25–29]. In fact, the betweenness of some node/gene reflects the direct and indirect relationship of the gene and known glioma related genes. Thus, the likelihood of genes with high betweenness to be related to glioma was higher than those with low betweenness. In view of this, we selected genes with betweenness greater than 0 as the candidate genes, which may be the novel glioma related genes with high probability. It is necessary to point out that the known glioma related genes were not included in the set of candidate genes.

### 2.4. Filtering Candidate Genes by Permutation Test

As described in Section 2.3, some candidate genes can be obtained by researching the shortest paths connecting any two known glioma related genes. However, some of them may be false positives, because some nodes/genes may easily receive a high betweenness due to their location in the weighted graph even if we randomly select genes in STRING as the known glioma related genes. To exclude these false discoveries, a permutation test should be executed as follows.

(I) 1,000 node/gene sets, denoted by *G*_1_, *G*_2_,…, *G*_1000_, were randomly selected in the weighted graph such that each of them had the same size of known glioma related gene set.
(II) For each candidate gene discovered in Section 2.3, calculate its betweenness on each set *G*_*i*_  (1 ≤ *i* ≤ 1000).
(III) Calculate the permutation FDR of each candidate gene *p* by
  (2) $$\begin{array}{c} \text{FDR}\left(p\right)=\frac{\sum_{i=1}^{1000}\delta_{i}}{1000}, \end{array}$$
  where *δ*_*i*_ was set to be 1 if the betweenness of *p* on *G*_*i*_ was larger than that of *p* on the known glioma related gene set; otherwise, it was set to be 0.

Obviously, small permutation FDR of one candidate gene implies that it is the true positive with high probability.

## 3. Results and Discussions

### 3.1. Candidate Genes

For the 77 genes mentioned in Section 2.1, we searched the shortest path connecting any two of them by Dijkstra algorithm. After calculating the betweenness of each node/gene in the weighted graph, 215 candidate genes with betweenness larger than zero were obtained. These 215 genes and their betweenness were available in Supplementary Material II. To exclude the false positives, the permutation test was conducted as described in Section 2.4. By (2), we can calculate the permutation FDR of each candidate gene. These values were also provided in Supplementary Material II. Since the likelihood of gene with small permutation FDR to be the actual glioma related gene is high, we set the threshold to be 0.05, that is, selecting genes with permutation FDRs lower than 0.05 among 215 candidate genes, thereby obtaining 67 genes, listed in Table 1. These genes were deemed to have strong relationship with glioma and further discussions were based on these genes.

### 3.2. Analysis of Enriched KEGG Pathways of Candidate Genes

As mentioned in Section 3.1, 67 candidate genes were obtained. To analyze the relationship of them and glioma, we employed DAVID (Database for Annotation, Visualization and Integrated Discovery) [30], a functional annotation tool to understand biological meaning behind large list of genes. 67 candidate genes comprised the input gene list of DAVID, thereby obtaining 9 KEGG pathways that were enriched by these 67 candidate genes. The detailed output of DAVID for KEGG pathway enrichment analysis was available as Supplementary Material III.

The top 5 pathways have the *P* value less than 0.05 which were discussed below. The most enriched pathway is hsa04360: axon guidance (“count” = 8). Among the 8 genes, 7 ephrins-related genes are enriched whose corresponding proteins include 4 members (EFNA3, EFNB1, EFNB2, and EFNB3) of the ephrins family and 3 members (EPHA1, EPHA4, and EPHA7) of the ephrins receptor subfamily. Eph receptor tyrosine kinases (Ephs) and ephrins (EPH) could navigate cells by controlling cell-cell adhesion and segregation [31]. In other words, with the function of axon guidance Ephs/EPH could regulate the invasion, neoangiogenesis, and metastasis of gliomas [32, 33]. Ding and his colleagues have identified several somatic mutations of Ephs especially EphA7 in lung cancer [34]. Although the close connection between Ephs/EPH and cancer has been reported, its pathogenic mechanism in gliomas is still unknown. The second pathway is hsa04510: focal adhesion (“count” = 7). As we know, infiltration of tumor cells and angiogenesis are critical for the growth of tumor. Zagzag et al. reported that focal adhesion kinase (FAK), highly associated with these biological processes, plays an important role in tumorigenesis of gliomas via enhancing the ability of infiltration and angiogenesis [35]. The FAK-related genes, like CTNNB1, VEGFA, KDR, and FLT4 enriched in this pathway, are always mutated or aberrantly expressed in various types of cancers [36, 37]. The third pathway is hsa04530: tight junction (“count” = 5). In the brain, the expression of the tight junction proteins is important for blood-brain tumor-barrier (BTB) permeability. Hence destruction of the tight junction could facilitate the development of gliomas by increasing BTB permeability [38, 39]. The fourth pathway is hsa04520: adherens junction (“count” = 4). Adherens junction is reported to be disordered in the glioblastoma and to affect the invasive behavior of GBM [40, 41]. The last significantly enriched pathway is hsa05200: pathways in cancer (“count” = 7). The result shows that a common mechanism is shared by the gliomas and other types of cancers. Although these significant enriched pathways have been reported to be related to gliomas more or less, our results might expand the avenues to explore new mechanisms in the tumorigenesis of gliomas.

### 3.3. Analysis of Enriched GO Terms Candidate Genes

In addition to KEGG pathway enrichment analysis, DAVID also provided the GO terms enrichment analysis of the 67 candidate genes, which were available in Supplementary Material IV.

It can be seen that 227 GO terms were enriched by these 67 genes. Top 10 Go terms sorted by *P* value are investigated and discussed as below. Among the top 10, 4 GO terms are biological process (BP) which included GO: 0007169: transmembrane receptor protein tyrosine kinase signaling pathway, GO: 0007167: enzyme linked receptor protein signaling pathway, GO: 0042127: regulation of cell proliferation, and GO: 0000904: cell morphogenesis involved in differentiation. From the results, we found that all these processes are connected with receptor-dependent signaling pathways. The cancer genome atlas (TCGA) group has revealed that the receptor tyrosine kinase (RTK) pathway was deregulated in 88% of the patients with glioblastoma [42]. After deregulation of the RTK, its downstream genes could function uncontrollably in the cellular proliferation and morphogenesis which are very pivotal for the growth of gliomas. In the top 10 GO terms, we also find 4 molecular function (MF) GO terms: GO: 0004714: transmembrane receptor protein tyrosine kinase activity, GO: 0005003: ephrin receptor activity, GO: 0046875: ephrin receptor binding, and GO: 0004713: protein tyrosine kinase activity. The MF classification also suggests the importance of RTK signaling pathways especially the Ephs/EPH pathway in the tumorigenesis of gliomas. As the previous reports, RTK pathways could regulate cell proliferation and migration which were indispensable for the development of gliomas [42, 43]. Besides BP and MF GO terms, 2 cellular component (CC) GO terms are also enriched in the top 10 GO terms: GO: 0044459: plasma membrane part and GO: 0005887: integral to plasma membrane. As we know, the transformation of cell membrane is necessary for the migration and invasion process during tumorigenesis of gliomas. Our results pave the way for understanding potential pathogenic mechanism of gliomas.

### 3.4. Analysis of Some Candidate Genes

Among the 67 genes, several genes are intriguing which may play pivotal role in tumorigenesis of glioma. This section gave the detailed discussion of some candidate genes.

VEGFA, also known as the vascular endothelial growth factor (VEGF), is a member of a large family of growth factors that also includes VEGFB, VEGFC, VEGFD, and placental growth factor (PLGF). VEGF is the only mitogen that specifically acts on endothelial cells and also a tumor angiogenesis factor in human glioma* in vivo* [44]. Knizetova et al. have demonstrated that the autocrine VEGF signaling is mediated via VEGFR2 (KDR), another gene in our list. They found that blockade of VEGFR2 would abrogate the VEFG-mediated enhancement of astrocytoma cell growth and viability [37]. In the* in vivo* level, Millauer et al. found that disrepair of VEGFR2/VEGF system in angiogenesis could prevent tumor growth in nude mice [45]. Another VEGF receptor found in our list is VEGFR3 (also known as FLT4). In contrast to VEGFR1/2, VEGFR3 does not bind VEGFA and mainly functions in lymphangiogenesis as a receptor of VEGFC and VEGFD [46, 47]. Jenny et al. reported that VEFGR3 was expressed in some tumor types such as haemangioblastoma and glioblastoma, despite their lack of lymphatic vessels [48]. Although the roles of VEGF signaling pathway in the tumorigenesis of glioma have been well studied, new findings have been explored in succession recently.

CTNNB1, with more famous name of catenin beta 1, encodes *β*-catenin protein which plays important roles in cellular morphogenesis, differentiation, and proliferation via regulating the Wnt signaling [49]. Yano et al. induced glioma in rat using N-ethyl-N-nitrosourea display aberrant nuclear accumulation of *β*-catenin in contrast to normal brains [50]. Moreover, Pu et al. found that the downregulation of *β*-catenin by siRNA could suppress malignant glioma cell growth [36]. To elucidate the connection between *β*-catenin and glioma, Liu and his colleagues performed a systemic research. They found a higher expression level of *β*-catenin in astrocytic glioma patients with high grade in comparison with the normal controls. Furthermore, they also illustrated that the overexpression of *β*-catenin may be an important contributing factor to glioma progression by means of facilitating proliferation and inhibiting apoptosis [51]. After Wnt pathway is activated, *β*-catenin accumulates and enters the nucleus where it can act as a coactivator for TCF/LEF-mediated transcription [52]. As the downstream of beta-catenin, LEF1, another gene in our list, also plays a crucial role in the Wnt signaling pathway. LEF1, with full name of lymphoid enhancer-binding factor 1, tends to be mutated in the tumors. Liu et al. have investigated that MiR-218 could reduce the invasiveness of glioblastoma cells by targeting LEF1 [53].

SRC, whose corresponding protein is a tyrosine-protein kinase, could play a pivotal role in the regulation of embryonic development and cell growth [54]. Besides functioning in the embryonic development, SRC could also regulate the tumorigenesis of various types of cancers like breast cancer, colon cancer, and brain cancer [55, 56]. Src protein always maintains an inactive state until its Y530 residue is dephosphorylated by protein tyrosine phosphatase-*α* [57]. Src could also be activated by direct binding of its SH2 and SH3 domains to intracellular proteins or activated tyrosine kinase growth factor receptors [58]. Stettner et al. have found elevated SRC activity in GBM compared with normal brain [59]. On the other hand, Lund et al. found that the infiltration of glioma reduced in Src-deficient mice [60]. It is reported that the increased SRC activity in GBM may be due to increased activation of cell surface growth factor receptors and integrins that activate SRC-family kinases (SFKs) rather than the amplification or mutation of SFK genes [42, 61].

## 4. Conclusion

In biomedicine and genomics, identification of disease genes is an important topic. This contribution proposed a graphic method to identify novel disease genes and the method was applied to glioma, one kind of cancers. The findings indicate that this method is quite effective. It is hopeful that the contribution can provide help for medical workers to discover effective treatments of glioma and give new insight to study various diseases.

## Supplementary Material

 The Supplementary Material contains four files. In detail, Supplementary Material I lists 77 glioma related genes; Supplementary Material II lists 215 candidate genes with betweenness greater than 0 and their permutation FDRs; Supplementary Material III lists KEGG enrichment results of 67 selected genes; Supplementary Material IV lists GO enrichment results of 67 selected genes.

## Figures and Tables

**Table 1:** Candidate genes with permutation FDR lower than 0.05.

Ensemble ID of candidate gene	Gene name	Betweenness	Permutation FDR
ENSP00000227638	PANX1	72	0
ENSP00000235310	MAD2L2	72	0
ENSP00000245323	EFNB2	335	0
ENSP00000258428	REV1	72	0
ENSP00000265727	ADAM22	72	0
ENSP00000281821	EPHA4	339	0
ENSP00000293831	EIF4A1	72	0
ENSP00000302719	KCNAB3	72	0
ENSP00000312697	DMAP1	88	0
ENSP00000329797	CADM1	72	0
ENSP00000335434	WDR20	72	0
ENSP00000341138	EPB41L3	72	0
ENSP00000351697	REV3L	72	0
ENSP00000354778	CNTNAP2	72	0
ENSP00000356150	MDM4	72	0
ENSP00000357177	ARHGEF11	142	0
ENSP00000361366	SFTPD	72	0
ENSP00000369218	RBM17	72	0
ENSP00000370119	SMN2	72	0
ENSP00000245304	RAP2A	72	0.001
ENSP00000275815	EPHA1	72	0.001
ENSP00000328511	KCNA4	72	0.001
ENSP00000377446	SUCLG1	210	0.001
ENSP00000399511	TNIK	72	0.001
ENSP00000229595	ASF1A	72	0.002
ENSP00000252699	ACTN4	72	0.002
ENSP00000263208	HIRA	72	0.002
ENSP00000263923	KDR	104	0.002
ENSP00000304169	PITX2	210	0.002
ENSP00000330633	CNTN2	72	0.002
ENSP00000276072	TAF1	72	0.003
ENSP00000295600	MITF	138	0.003
ENSP00000360157	FOXD3	6	0.003
ENSP00000264010	CTCF	72	0.004
ENSP00000271628	SF3B4	75	0.004
ENSP00000350941	SRC	421	0.004
ENSP00000361125	VEGFA	164	0.004
ENSP00000226091	EFNB3	67	0.006
ENSP00000358309	EPHA7	2	0.006
ENSP00000358918	SUFU	72	0.007
ENSP00000316879	EIF4G1	72	0.011
ENSP00000260653	SIX3	2	0.012
ENSP00000352516	DNMT1	90	0.016
ENSP00000358716	DDX20	72	0.017
ENSP00000357393	EFNA3	50	0.018
ENSP00000341680	DTNBP1	66	0.02
ENSP00000344456	CTNNB1	607	0.02
ENSP00000297904	FIGF	2	0.021
ENSP00000265165	LEF1	134	0.022
ENSP00000347948	TNFRSF14	68	0.023
ENSP00000288986	NCK1	78	0.027
ENSP00000261937	FLT4	2	0.029
ENSP00000333919	BTLA	68	0.03
ENSP00000332549	GRIN2A	58	0.032
ENSP00000376765	PIAS3	4	0.033
ENSP00000361818	SDC4	1	0.035
ENSP00000386165	CEBPD	38	0.035
ENSP00000348307	SIRPA	34	0.036
ENSP00000344666	NF2	1	0.037
ENSP00000219255	PARD6A	72	0.038
ENSP00000204961	EFNB1	5	0.039
ENSP00000172229	NGFR	72	0.043
ENSP00000344115	CDH5	24	0.043
ENSP00000405041	POU5F1	6	0.045
ENSP00000360532	CDC5L	6	0.046
ENSP00000295897	ALB	72	0.047
ENSP00000340944	PTPN11	112	0.047

## References

[1] Louis DN, Ohgaki H, Wiestler OD (2007). The 2007 WHO classification of tumours of the central nervous system. *Acta Neuropathologica*.

[2] Dolecek TA, Propp JM, Stroup NE, Kruchko C (2012). CBTRUS statistical report: primary brain and central nervous system tumors diagnosed in the United States in 2005–2009. *Neuro-Oncology*.

[3] Louis DN (1994). The p53 gene and protein in human brain tumors. *Journal of Neuropathology and Experimental Neurology*.

[4] Henson JW, Schnitker BL, Correa KM (1994). The retinoblastoma gene is involved in malignant progression of astrocytomas. *Annals of Neurology*.

[5] Guha A, Feldkamp MM, Lau N, Boss G, Pawson A (1997). Proliferation of human malignant astrocytomas is dependent on Ras activation. *Oncogene*.

[6] Knobbe CB, Trampe-Kieslich A, Reifenberger G (2005). Genetic alteration and expression of the phosphoinositol-3-kinase/Akt pathway genes *PIK3CA* and *PIKE* in human glioblastomas. *Neuropathology and Applied Neurobiology*.

[7] Rinkenbaugh AL, Baldwin AS (2011). Monoallelic deletion of NFKBIA in glioblastoma: when less is more. *Cancer Cell*.

[8] Chen L, Zeng WM, Cai YD, Feng KY, Chou KC (2012). Predicting anatomical therapeutic chemical (ATC) classification of drugs by integrating chemical-chemical interactions and similarities. *PLoS ONE*.

[9] Yamanishi Y, Araki M, Gutteridge A, Honda W, Kanehisa M (2008). Prediction of drug-target interaction networks from the integration of chemical and genomic spaces. *Bioinformatics*.

[10] Yamanishi Y, Kotera M, Kanehisa M, Goto S (2010). Drug-target interaction prediction from chemical, genomic and pharmacological data in an integrated framework. *Bioinformatics*.

[11] Chen L, Lu J, Luo X, Feng K-Y (2014). Prediction of drug target groups based on chemical-chemical similarities and chemical-chemical/protein connections. *Biochimica et Biophysica Acta: Proteins and Proteomics*.

[12] Padhy B, Gupta Y (2011). Drug repositioning: re-investigating existing drugs for new therapeutic indications. *Journal of Postgraduate Medicine*.

[13] Chen L, Lu J, Zhang N, Huang T, Cai Y-D (2014). A hybrid method for prediction and repositioning of drug Anatomical Therapeutic Chemical classes. *Molecular BioSystems*.

[14] Chen L, Zeng W-M, Cai Y-D, Huang T (2013). Prediction of metabolic pathway using graph property, chemical functional group and chemical structural set. *Current Bioinformatics*.

[15] Ma H-W, Zeng A-P (2003). The connectivity structure, giant strong component and centrality of metabolic networks. *Bioinformatics*.

[16] Dale JM, Popescu L, Karp PD (2010). Machine learning methods for metabolic pathway prediction. *BMC Bioinformatics*.

[17] Chen L, Li B-Q, Feng K-Y (2013). Predicting biological functions of protein complexes using graphic and functional features. *Current Bioinformatics*.

[18] Gao YF, Chen L, Cai YD, Feng KY, Huang T, Jiang Y (2012). Predicting metabolic pathways of small molecules and enzymes based on interaction information of chemicals and proteins. *PLoS ONE*.

[19] Sharan R, Ulitsky I, Shamir R (2007). Network-based prediction of protein function. *Molecular Systems Biology*.

[20] Ng KL, Ciou JS, Huang CH (2010). Prediction of protein functions based on function-function correlation relations. *Computers in Biology and Medicine*.

[21] Bogdanov P, Singh AK (2010). Molecular function prediction using neighborhood features. *IEEE-ACM Transactions on Computational Biology and Bioinformatics*.

[22] Gao P, Wang QP, Chen L, Huang T (2012). Prediction of human genes' regulatory functions based on proteinprotein interaction network. *Protein and Peptide Letters*.

[23] Jensen LJ, Kuhn M, Stark M (2009). STRING 8—a global view on proteins and their functional interactions in 630 organisms. *Nucleic Acids Research*.

[24] Gormen TH, Leiserson CE, Rivest RL, Stein C (1990). *Introduction to Algorithms*.

[25] Davis J, Goadrich M The relationship between precision-recall and ROC curves.

[26] Bunescu R, Ge R, Kate RJ (2005). Comparative experiments on learning information extractors for proteins and their interactions. *Artificial Intelligence in Medicine*.

[27] Johnson DE, Wolfgang GHI (2000). Predicting human safety: screening and computational approaches. *Drug Discovery Today*.

[28] Li B-Q, Niu B, Chen L (2013). Identifying chemicals with potential therapy of HIV based on protein-protein and protein-chemical interaction network. *PLoS ONE*.

[29] Zhang J, Jiang M, Yuan F, Feng KY, Cai YD (2013). Identification of age-related macular degeneration related genes by applying shortest path algorithm in protein-protein interaction network. *BioMed Research International*.

[30] Huang DW, Sherman BT, Lempicki RA (2009). Systematic and integrative analysis of large gene lists using DAVID bioinformatics resources. *Nature Protocols*.

[31] Klein R (2004). Eph/ephrin signaling in morphogenesis, neural development and plasticity. *Current Opinion in Cell Biology*.

[32] Janes PW, Adikari S, Lackmann M (2008). Eph/ephrin signalling and function in oncogenesis: Lessons from embryonic development. *Current Cancer Drug Targets*.

[33] Pasquale EB (2010). Eph receptors and ephrins in cancer: bidirectional signalling and beyond. *Nature Reviews Cancer*.

[34] Ding L, Getz G, Wheeler DA (2008). Somatic mutations affect key pathways in lung adenocarcinoma. *Nature*.

[35] Zagzag D, Friedlander DR, Margolis B (2000). Molecular events implicated in brain tumor angiogenesis and invasion. *Pediatric Neurosurgery*.

[36] Pu P, Zhang Z, Kang C (2009). Downregulation of Wnt2 and *β*-catenin by siRNA suppresses malignant glioma cell growth. *Cancer Gene Therapy*.

[37] Knizetova P, Ehrmann J, Hlobilkova A (2008). Autocrine regulation of glioblastoma cell cycle progression, viability and radioresistance through the VEGF-VEGFR2 (KDR) interplay. *Cell Cycle*.

[38] Gu YT, Xue YX, Wei XY, Zhang H, Li Y (2011). Calcium-activated potassium channel activator down-regulated the expression of tight junction protein in brain tumor model in rats. *Neuroscience Letters*.

[39] Xie H, Xue YX, Liu LB, Liu YH (2010). Endothelial-monocyte-activating polypeptide II increases blood-tumor barrier permeability by down-regulating the expression levels of tight junction associated proteins. *Brain Research*.

[40] Nikuseva-Martic T, Beros V, Pecina-Slaus N, Pecina HI, Bulic-Jakus F (2007). Genetic changes of CDH1, APC, and CTNNB1 found in human brain tumors. *Pathology: Research and Practice*.

[41] Perego C, Vanoni C, Massari S (2002). Invasive behaviour of glioblastoma cell lines is associated with altered organisation of the cadherin-catenin adhesion system. *Journal of Cell Science*.

[42] The Cancer Genome Atlas Research Network (2008). Comprehensive genomic characterization defines human glioblastoma genes and core pathways. *Nature*.

[43] Rao SA, Arimappamagan A, Pandey P (2013). miR-219-5p inhibits receptor tyrosine kinase pathway by targeting EGFR in glioblastoma. *PLoS ONE*.

[44] Plate KH, Breier G, Weich HA, Risau W (1992). Vascular endothelial growth factor is a potential tumour angiogenssis factor in human gliomas in vivo. *Nature*.

[45] Millauer B, Shawver LK, Plate KH, Risau W, Ullrich A (1994). Glioblastoma growth inhibited in vivo by a dominant-negative Flk-1 mutant. *Nature*.

[46] Koch S, Tugues S, Li X, Gualandi L, Claesson-Welsh L (2011). Signal transduction by vascular endothelial growth factor receptors. *The Biochemical Journal*.

[47] Karkkainen MJ, Petrova TV (2000). Vascular endothelial growth factor receptors in the regulation of angiogenesis and lymphangiogenesis. *Oncogene*.

[48] Jenny B, Harrison JA, Baetens D (2006). Expression and localization of VEGF-C and VEGFR-3 in glioblastomas and haemangioblastomas. *Journal of Pathology*.

[49] Anastas JN, Moon RT (2013). WNT signalling pathways as therapeutic targets in cancer. *Nature Reviews Cancer*.

[50] Yano H, Hara A, Shinoda J (2000). Immunohistochemical analysis of *β*-catenin in N-ethyl-N-nitrosourea- induced rat gliomas: implications in regulation of angiogenesis. *Neurological Research*.

[51] Liu X, Wang L, Zhao S, Ji X, Luo Y, Ling F (2011). *β*-catenin overexpression in malignant glioma and its role in proliferation and apoptosis in glioblastma cells. *Medical Oncology*.

[52] Polakis P (2000). Wnt signaling and cancer. *Genes & Development*.

[53] Liu Y, Yan W, Zhang W (2012). MiR-218 reverses high invasiveness of glioblastoma cells by targeting the oncogenic transcription factor LEF1. *Oncology Reports*.

[54] Nada S, Yagi T, Takeda H (1993). Constitutive activation of Src family kinases in mouse embryos that lack Csk. *Cell*.

[55] Zhang S, Huang WC, Zhang L, Zhang C, Lowery FJ (2013). SRC family kinases as novel therapeutic targets to treat breast cancer brain metastases. *Cancer Research*.

[56] Chen J, Elfiky A, Han M, Chen C, Saif MW (2014). The Role of Src in colon cancer and its therapeutic implications. *Clinical Colorectal Cancer*.

[57] Egan C, Pang A, Durda D, Cheng H, Wang JH, Fujita DJ (1999). Activation of Src in human breast tumor cell lines: elevated levels of phosphotyrosine phosphatase activity that preferentially recognizes the Src carboxy terminal negative regulatory tyrosine 530. *Oncogene*.

[58] Yeatman TJ (2004). A renaissance for SRC. *Nature Reviews Cancer*.

[59] Stettner MR, Wang W, Nabors LB (2005). Lyn kinase activity is the predominant cellular Src kinase activity in glioblastoma tumor cells. *Cancer Research*.

[60] Lund CV, Nguyen MT, Owens GC (2006). Reduced glioma infiltration in Src-deficient mice. *Journal of Neuro-Oncology*.

[61] Ahluwalia MS, de Groot J, Liu WM, Gladson CL (2010). Targeting SRC in glioblastoma tumors and brain metastases: rationale and preclinical studies. *Cancer Letters*.

